# Naming is not explaining: future directions for the “cognitive reserve” and “brain maintenance” theories

**DOI:** 10.1186/s13195-018-0365-z

**Published:** 2018-04-02

**Authors:** Jonna Nilsson, Martin Lövdén

**Affiliations:** Aging Research Center, Department of Neurobiology, Care Sciences, and Society, Karolinska Institutet and Stockholm University, Gävlegatan 16, 113 30 Stockholm, Sweden

**Keywords:** Cognitive aging, Dementia, Cognitive reserve, Brain maintenance

## Abstract

Contemporary imaging measures of the human brain explain less than half of the differences in cognitive functioning and change among older adults. Researchers have advanced several theories and concepts to guide research that aims to better explain these individual differences in cognitive aging. Taking the fundamental measurement model in the empirical sciences as a starting point, we here scrutinize two such complementary theories, brain maintenance and cognitive reserve, in an attempt to clarify these theories, gauge their usefulness, and identify ways in which they can be further developed. We demonstrate that, although both theories are highly useful for spawning theorizing and empirical work, they can be further developed by detailing the theoretical and operational definitions of the concepts that they propose. We propose a few ways forward in these directions.

## Background

Science has a long way to go in mapping cognition to the brain. People differing in cognitive ability may have identical brains, as currently measurable. Individuals with different brains may display identical cognitive functioning. In fact, contemporary imaging measures of the human brain cannot explain much more than 40% of the differences in cognitive functioning and change among older adults [1–3]. Several theories and concepts have been proposed for guiding research that aims to increase our understanding of why individuals perform differentially, age with more or less success, and display variable resiliency to adversity [4–11]. Using the fundamental measurement model in the empirical sciences as a framework, we here scrutinize two such theories, brain maintenance [8] and cognitive reserve [12–14], in an attempt to clarify their meaning, gauge their usefulness, and identify ways in which they can be further developed.

### Concepts and theories in empirical science

A concept can be defined either as ideal phenomena (e.g., fish or brain) or as generally agreed-upon phenomena (e.g., center of mass or intelligence) that exist only in the minds of humans, including researchers. Although concepts are abstract and do not have a physical or concrete existence, they can be manifested in reality. For example, the concept of fish is manifested in particular specimens of fish, and although the concept of intelligence is abstract, it is manifested in human behavior. Concepts are the building blocks of hypotheses, which essentially are collections of concepts and their associations (e.g., concept “brain” causes concept “intelligence”). A scientific theory emerges when these hypotheses become substantiated.

Owing to their abstract nature, concepts can have fuzzy theoretical definitions. Theories consisting of such vague concepts may nevertheless be useful by functioning as research programs. Such programs may be considered scientific and useful if they can be developed to make novel and testable predictions, and especially if these predictions can be confirmed [15]. Initially vague concepts may also be useful because they provoke productive efforts to specify and refine their meaning. Scientific theorizing includes a fair share of such efforts. For example, Einstein [16] could develop the theory of special relativity by strictly defining things such as length and simultaneity—concepts that were previously taken for granted. Thus, although stringent theoretical definitions of concepts move theory forward, an initially vague concept can be useful in many ways.

In addition to theoretical stringency, operational definition is a crucial part of clarifying scientific concepts [17]. As opposed to a theoretical definition, which is abstract and conceptual (e.g., episodic memory is memory for personally experienced events [18]), an operational definition is a down-to-earth description of how a concept is manifested in reality and thereby how it is measured (e.g., numbered of recalled words in a memory test). In fact, certain traditions in the philosophy of science would dictate that only an operationally defined concept constitutes a scientific construct [19]. Operationally defining concepts is critical because it makes theories empirically testable: a necessary but not sufficient condition for rendering theories empirically falsifiable. In turn, it is typically assumed that a theory must be falsifiable to qualify as scientific [20]. Thus, researchers in empirical sciences such as the social sciences, the behavioral sciences, and medicine generally agree that operational definitions are what make theories scientific.

**Fig. 1:**
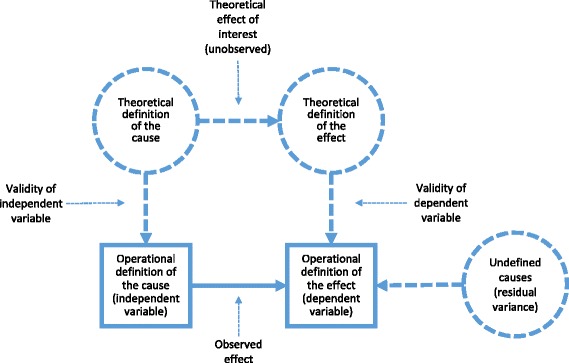
Basic scientific model in empirical science. Theories and hypotheses are collections of theoretically (conceptually) defined causes and effects (scientific constructs). The effect (association between scientific constructs) that a hypothesis or theory predicts cannot be directly observed but can only be inferred from the association between the operationally defined variables. The quality of this inference relies on the validity of the operational definitions. This so-called construct validity cannot be directly observed. Due to inadequate operational definitions or inadequate theory, the association between the operationally defined variables is typically less than perfect, leaving unexplained, or residual, variance (i.e., theoretically and operationally undefined causes) in the dependent variable. The model may be further refined (e.g., by explicitly considering the dimension of time, incorporating latent variable modeling, and allowing for error variance), but these refinements would not affect the major conclusions we draw in this review. Circles represent unobserved variables (scientific constructs) and squares represent observed variables (data). Thick arrows represent observed associations and dashed lines represent unobserved associations

Researchers in empirical science thus draw inferences from observed associations between observed variables to shed light on what they really care about: the unobserved associations between unobserved theoretical constructs. The quality of these inferences obviously relies on the quality of the operational definitions; that is, to what degree that which is measured actually reflects the intended scientific construct (i.e. construct validity [19]). For example, is the number of recalled words on a memory test reflecting episodic memory and not working memory? Unfortunately, construct validity is not possible to directly inspect, which is troublesome because inadequate operational definitions can bias theoretical inferences. Inadequate operational definitions of a cause will also result in unexplained variance (i.e., a residual) in the effect of interest, although the theory would be accurate in the sense that the proposed cause is the only source of the effect. This will be the case because the measured cause may only cover select aspects of the theoretical cause. For example, a researcher may postulate a hypothesis that individual differences in the brain fully account for differences in intelligence, but because researchers are not measuring all relevant brain differences there will remain unexplained differences in intelligence. Therefore, with less-than-perfect operational definitions (i.e., suboptimal construct validity), which are the rule rather than the exception, it is impossible to determine whether unexplained variance in the dependent variable is due to inadequate theory or inadequate measurement. Figure 1 illustrates this basic scientific model in the empirical sciences.

We take this basic model as the starting point of a theoretical analysis of two terms frequently employed in research on cognitive aging and dementia: brain maintenance [8] and cognitive reserve [12–14]. We think that critically evaluating the use of these terms is crucial to the advancement of the fields of normal and pathological aging, and that the fundamental measurement model in the empirical sciences provides the appropriate evaluative framework.

### Brain maintenance

At the heart of the term brain maintenance is the notion that between-person differences in how well preserved people’s brains are as they age can explain between-person differences in within-person changes in cognitive ability in aging [8]. In other words, an older brain that performs more like it did in younger age is a brain that it is well preserved. That is, the less the brain changes with age (structurally, chemically, and functionally), the less cognitive ability will decline; and, conversely, the more the brain changes, the more cognitive ability will decline. Successful cognitive aging is thus about maintaining the brain in the same shape as when it was younger. Inherent in the notion of brain maintenance is thus a positive association between two theoretically defined constructs. The first is a construct of brain change (or, conversely, preservation of the brain) that we may theoretically define as any change in performance-relevant brain properties. The second is a construct of change in cognitive ability (or, conversely, stability of cognitive ability) that we may theoretically define as change in the difficulty of a defined class of mental tasks that an individual can master [21]. The term maintenance refers to the idea that certain putative factors, such as physical activity, can help maintain the youth of the brain by reducing overall brain change and by more active neural repair processes, and thereby reduce change in cognitive ability.

**Fig. 2:**
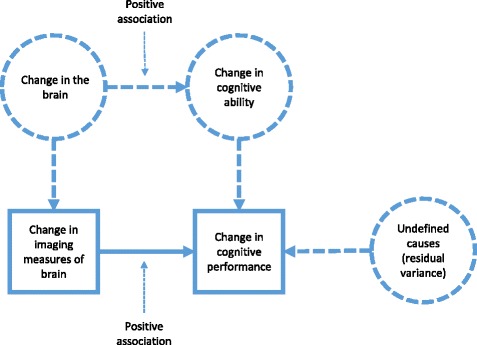
Graphical description of brain maintenance theory [8]. The theory proposes that a construct of brain change, theoretically defined as any task-related change in the brain in aging (i.e., lack of preservation), causes aging-related decrements in a cognitive ability construct. Brain change is operationally defined as within-person change in neuroimaging measures of brain structure (e.g., volume, white matter microstructure, and vascular pathology) and brain function over time. Change in cognitive ability is manifested as change in cognitive performance. The theory proposes no constructs other than brain change to explain changes in cognitive ability. In addition to the core of the theory illustrated, the theory also proposes that factors that may play a role in a resilience process, such as education or physical activity, or shape changes in cognitive ability by minimizing brain change. Circles represent unobserved variables (scientific constructs) and squares represent observed variables (data). Thick arrows represent observed associations and dashed lines represent unobserved associations

The purpose of the first presentation of brain maintenance theory [8] was more to advance a theory in the form of research program than to offer a stringent theory with specific concepts. However, we can quite easily generate some examples of operational definitions of the proffered theoretical constructs. Brain change can be manifested in measures of within-person changes over time; for example, in neuroimaging measures of brain structure (e.g., volume, cortical thickness, white matter microstructure, amyloid burden) and brain function (e.g., the blood oxygen-level dependent signal). Changes in cognitive ability can be manifested in longitudinal changes in measures of cognitive performance. The predicted association between the theoretical constructs appears conceptually justified (i.e., brain changes cause changes in cognitive ability) and has some empirical support, as some changes in imaging measures of the human brain have been associated with aging-related changes in cognitive performance [2, 8, 22]. Thus, brain maintenance is a theory, as it describes a relatively well-substantiated hypothesis of an association between two theoretical constructs. Figure 2 depicts this theory using the same graphical notation as in the previous section.

Brain maintenance theory thus focuses on explaining change in cognitive ability in old age rather than on explaining older individuals’ absolute level of cognitive performance (and thus not on whether an individual passes a diagnosis threshold for dementia). Thus, according to the theory of brain maintenance, successful aging is defined as minimal change in cognitive ability in aging, independent of absolute functional level. To predict the level of cognitive ability, the theory implicitly assumes another construct: the level of brain integrity measured concurrently with cognitive ability or, alternatively, the level of brain integrity measured in the individual when younger (which has been discussed under the term “brain reserve” in, e.g., [23]) plus the change in brain integrity thereafter (see also [24]). Importantly, brain maintenance theory is falsifiable because it specifies a positive association between brain change and cognitive change in aging. A negative association between some aspect of brain change and change in cognitive performance (i.e., finding that a brain that changed in some way with age performs better than a brain that stayed the same) would mean that additional constructs are needed to fully explain age-related decline in cognitive performance.

Brain maintenance theory is a useful research program that has proven productive in generating much empirical and theoretical work. It is useful because the theory provokes productive efforts to specify and refine the meaning of brain change—thus guiding researchers to the task of finding brain correlates of cognitive decline. Arguably, the model behind brain maintenance (i.e., brain change is positively associated with cognitive change) is also the model that many researchers have worked with in an implicit way for a long time. The proposal of brain maintenance theory makes this model explicit. The challenge for brain maintenance theory as a more stringent theory, beyond its useful function as a research program, is the vague and comprehensive theoretical definition of brain change, which makes all operational definitions inadequate. Unfortunately, this inadequate operational definition can easily be mistaken for an inadequate theory, such that a failure to fully explain between-person differences in cognitive ability change (or level) in older adults with changes in (or level of) various neuroimaging measures falsifies brain maintenance theory and mandates additional explanatory constructs. As opposed to indicating a failing theory, it more likely indicates that the researcher has failed to measure all aspect of brain change that are relevant to explaining change in cognitive performance.

As an example, assume that the amyloid burden of two individuals is identical but that one individual is declining fast in cognitive performance and receives a dementia diagnosis, whereas the other is not declining much in performance and has no dementia diagnosis. In this situation, it seems tempting to conclude that between-person differences in brain change cannot explain cognitive change and that additional theoretical causes must be postulated. However, this inference relies on the validity of the operational definition of the brain change construct, and amyloid burden can only be said to capture a small part of this theoretical construct. For example, the high-functioning individual may have accumulated fewer vascular injuries or any number of other unknown, unmeasured, or imperfectly measured neurodegenerative alterations, perhaps because of advantageous lifestyle habits. Thus, the presence of residual variability in level of or change in functioning after accounting for select aspects of age-related brain pathology does not falsify the maintenance theory. In other words, there is no need for constructs other than brain change just because a particular operational definition of brain change fails to account for all of the variance in change in cognitive performance. This is because several aspects of brain integrity determine functioning and dementia diagnosis in old age, including those that have not been observed in this particular study or that have not yet been discovered.

It is therefore evident that the broad and unspecific theoretical definition of brain change that makes brain maintenance theory useful as a research program necessarily means that any operational definition of brain change will fall short and have limited construct validity. In practice, this means that proponents of brain maintenance theory can always explain failures to account for all cognitive change with unmeasured aspects of brain change. The consequence of this is that brain maintenance theory cannot be falsified by failures to account for changes in cognitive performance in older adults. In other words, because of an insufficient operational definition of brain change, theoretically unspecified causes cannot be distinguished from operationally undefined causes. The natural way forward is thus to use brain maintenance theory as an overarching research program to propose more refined definitions of brain change. It is these more specific theories, with their associated theoretical and operational definitions, that we should test by investigating their potential for accounting for variance in cognitive aging. That is, we should not confuse the research program of brain maintenance with its more specific instantiations. Such more specific theories are probably better seen as related theories under the general umbrella of the research program of brain maintenance. Examples include the dopamine theory [25] and the white matter (disconnection) theory [26, 27] of cognitive aging—just to mention a few. With a specific definition of brain change, it is also easy to extend the analyses to examine putative factors, such as physical activity, that could help to maintain the youth of the brain (e.g., [28]).

### Cognitive reserve

The cognitive reserve concept is defined theoretically as those between-person differences in how individuals process cognitive tasks that modulate how susceptible individuals are to the negative effects of brain change on cognitive ability [4, 14]. The concept therefore encompasses compensatory changes in cognitive processing in response to negative brain changes as a result of aging or disease as well as individual differences in how tasks are processed that exist before negative brain changes occur [4, 13, 29]. In addition to denoting this concept, the term cognitive reserve is also used to describe the theory postulating that this concept explains differences in cognitive performance in aging and that factors such as education can cause differences between individuals in their cognitive reserve.

Cognitive reserve is a concept that has been proposed to account for the difference between observed cognitive performance and the cognitive performance expected in an individual with a given degree of neuropathology [4, 12–14]. In other words, the cognitive reserve concept and the brain change concept of the brain maintenance theory are complementary. Although the cognitive reserve concept was proposed before the explicated brain maintenance theory, it appears to have emerged from a perceived insufficiency of measures of brain change to fully explain between-person differences in cognitive performance. That is, the then implicit model of brain maintenance was perceived as insufficient.

**Fig. 3:**
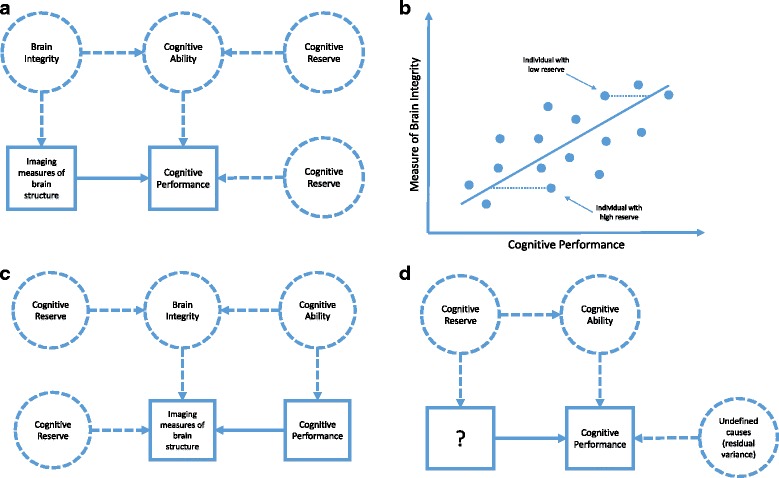
Approaches to defining cognitive reserve. **a** Theoretical and measurement model behind attempts to operationally define cognitive reserve as the variance in performance that is not explained by brain integrity. In this approach, the concept of cognitive reserve has no direct operational definition because it is not manifested in observable variables. **b** Difference between predicted and observed cognitive performances that have formed the attempts (also depicted in **a**) to measure individuals’ cognitive reserve. **c** Theoretical and measurement model behind attempts to operationally define cognitive reserve as the variance in brain integrity not predicted by cognitive performance. **d** A sound model of cognitive reserve that is likely to generate fruitful research if “?” can be replaced by a definition of measurement (i.e., if the cognitive reserve concept can be operationally defined). Squares represent observed variables (i.e., real stuff) and circles represent latent, unobserved concepts. Thick arrows represent observed associations and dashed lines represent unobserved associations

The proposal of the cognitive reserve theory has inspired an enormous mass of empirical work and provoked productive efforts to refine the cognitive reserve concept and related constructs. As such, the cognitive reserve proposal has proven extremely useful as a research program. The main challenge for refinement of the theory has been the difficulties with operationally defining the cognitive reserve concept. Paradoxically, the broad concepts of brain integrity and cognitive ability, rather than the theoretical definition of cognitive reserve in itself, have underpinned many attempts at operational definitions of the cognitive reserve concept. Brain integrity is, in these attempts, commonly operationally defined as some imaging measure of brain structure (e.g., gray matter volume and white matter microstructure) and cognitive ability by measures of cognitive performance. Cognitive ability is then explained as the sum of brain integrity and cognitive reserve. A serious challenge to this approach is that the cognitive reserve concept is not manifested in any observed (i.e., directly measured) aspect of reality but is instead commonly defined as the difference between observed cognitive performance and measures of brain integrity (e.g., [3, 30, 31]). Figure 3a clarifies the model behind this operational definition of cognitive reserve. An individual’s cognitive reserve is therefore determined solely by that individual’s deviation from the cognitive score predicted by the selected measures of brain integrity (see Fig. 3b). That is, the measure of cognitive reserve is the residual variance not accounted for by some measures of brain integrity.

The lack of an operational definition is unsatisfactory because it means, paradoxically, that the measure of the cognitive reserve concept does not depend on the construct validity of the concept itself, but on the validity of the operational definition of brain integrity. As discussed in the section on brain maintenance, variance in cognitive performance that is not explained by brain measures could point to the need for additional concepts to explain performance. However, it may also just reflect inadequate operational definitions of brain integrity. In other words, the presence of residual variance in cognitive performance after accounting for changes in some measure of the brain does not indicate the need for any other concept than brain change for explaining cognitive performance. In effect, in this way of operationally defining the cognitive reserve concept, better and more measures of brain integrity (e.g., adding amyloid imaging to MR imaging of gray matter volume and white matter microstructure) will affect the operational definition not only of brain integrity but also of cognitive reserve. The residual measure proposed to measure cognitive reserve will be independent of the measures of brain integrity, but this does not mean that it is independent of the theoretical construct of brain integrity unless the construct validity of brain integrity is perfect. Thus, this way to operationally define the cognitive reserve concept does not stand on its own feet and is therefore not adequate.

Although alternative operational definitions of cognitive reserve have been proposed, none has been satisfactory. For example, defining cognitive reserve as the variance in some brain measure that cannot be explained by cognitive performance (e.g., [32]) also does not qualify as an operational definition as it does not manifest in reality. The explicit model underlying this latter approach is depicted in Fig. 3c. Moreover, as is evident in Fig. 3c, this approach is based on a theoretical model stating that differences in cognitive ability and cognitive reserve causally influence brain integrity, which seems questionable for conceptual reasons.

Another attempt at specifying an operational definition of the cognitive reserve concept has been to aggregate variables that contribute to socioeconomic status and simply name the resulting factor cognitive reserve. Such attempts are also misguided because these variables are not manifestations of cognitive reserve, but rather potential causes of differences in cognitive reserve [33]. For example, theory does not propose that cognitive reserve causes more educational attainment but instead that educational attainment leads to more cognitive reserve. In other words, the indicators of the latent (unobserved) construct are not reflective but formative and are therefore entirely uninformative of the latent construct [33].

In sum, the concept of cognitive reserve currently has no operational definition. In a sound model, cognitive reserve would affect cognitive ability, but to measure cognitive reserve there would be no need for defining a brain integrity construct (Fig. 3d). That is, the cognitive reserve concept must the operationally defined. Only then can the associations that the cognitive reserve theory proposes, such as that education may increase cognitive reserve and that changes in brain integrity may induce compensatory strategies, be investigated.

Operationally defining the cognitive reserve concept probably requires a theoretical definition more thorough than those that currently exist. The theoretical definition of cognitive reserve as differences in how individuals process cognitive tasks (e.g., cognitive strategies), as compared to how well they perform in the same task, does provide a starting point for further refining the concept and developing ways of operationally defining the concept. Here we suggest that certain cognitive strategies, some of which may be measurable (e.g., mnemonic strategies [34, 35]), may be less sensitive to age-related brain change than others. Individuals who use such strategies can be said to possess greater reserve; their performance would be less affected by a particular brain change [4]. Operationally defining the cognitive reserve concept with measures of the qualitative way in which individuals approach cognitive tasks provides a direct operational definition of the cognitive reserve concept, such that its explanatory potential can be directly assessed. Along these lines, it may also be possible to develop operational definitions of this theoretical definition of cognitive reserve with differences in functional measure of the brain that exist despite identical cognitive task performance [29, 36].

## Conclusions

In science, there are many examples of concepts that were imagined before the precise theoretical definitions and measurements of these concepts were developed. In the case of brain maintenance and cognitive reserve theories, the vagueness of theoretical and operational definitions has provoked productive efforts to refine their meaning. However, as we have shown, none of the theories currently propose adequate operational definitions of their theoretical concepts. They function more as useful research programs than as stringent scientific theories. To avoid the (nominal) fallacy of thinking that naming is explaining, researchers must intensify efforts to operationally define the theoretical concepts so that their explanatory potential can be directly investigated.
